# Intra-abdominal infections survival guide: a position statement by the Global Alliance For Infections In Surgery

**DOI:** 10.1186/s13017-024-00552-9

**Published:** 2024-06-08

**Authors:** Massimo Sartelli, Philip Barie, Vanni Agnoletti, Majdi N. Al-Hasan, Luca Ansaloni, Walter Biffl, Luis Buonomo, Stijn Blot, William G. Cheadle, Raul Coimbra, Belinda De Simone, Therese M. Duane, Paola Fugazzola, Helen Giamarellou, Timothy C. Hardcastle, Andreas Hecker, Kenji Inaba, Andrew W. Kirkpatrick, Francesco M. Labricciosa, Marc Leone, Ignacio Martin-Loeches, Ronald V. Maier, Sanjay Marwah, Ryan C. Maves, Andrea Mingoli, Philippe Montravers, Carlos A. Ordóñez, Miriam Palmieri, Mauro Podda, Jordi Rello, Robert G. Sawyer, Gabriele Sganga, Pierre Tattevin, Dipendra Thapaliya, Jeffrey Tessier, Matti Tolonen, Jan Ulrych, Carlo Vallicelli, Richard R. Watkins, Fausto Catena, Federico Coccolini

**Affiliations:** 1 Department of Surgery, Macerata Hospital, Via Santa Lucia 2, Macerata, 62100 Italy; 2 Department of Surgery, Weill Cornell Medicine, New York, NY USA; 3 Anesthesia and Intensive Care Unit, Bufalini Hospital - AUSL della Romagna, Cesena, Italy; 4 Department of Internal Medicine, University of South Carolina School of Medicine, Columbia, SC USA; 5 Department of General and Emergency Surgery, Fondazione IRCCS San Matteo, Pavia, Italy; 6 Division of Trauma and Acute Care Surgery, Scripps Clinic Medical Group, La Jolla, CA USA; 7 Emergency, Urgency and Trauma Surgery, School of Medicine, University of Buenos Aires, Buenos Aires, Argentina; 8 Department of Internal Medicine and Pediatrics, Faculty of Medicine and Health Sciences, Ghent University, Ghent, Belgium; 9 Department of Surgery, School of Medicine, University of Louisville, Louisville, KY USA; 10 Comparative Effectiveness and Clinical Outcomes Research Center - CECORC - Riverside University Health System, Moreno Valley, CA USA; 11 Department of Surgery, Loma Linda University School of Medicine, Loma Linda, CA USA; 12 Department of Surgery, Rimini “Infermi” Hospital, Rimini, Italy; 13 Department of Surgery, Medical City Plano, Plano, TX USA; 14 First Department of Internal Medicine-Infectious Diseases, Hygeia General Hospital, Athens, Greece; 15 Department of Surgical Sciences, Nelson R Mandela School of Clinical Medicine, University of KwaZulu-Natal, and Inkosi Albert Luthuli Central Hospital, Durban, South Africa; 16 Department of General and Thoracic Surgery, University Hospital of Giessen, Giessen, Germany; 17 Department of Surgery, University of Southern California, Los Angeles, CA USA; 18 Department of Surgery and Critical Care Medicine, University of Calgary, Foothills Medical Centre, Calgary, AB Canada; 19 Global Alliance for Infections in Surgery, Macerata, Italy; 20 Department of Anaesthesia and Intensive Care Unit, AP-HM, Aix-Marseille University, North Hospital, Marseille, France; 21 Department of Intensive Care Medicine, Multidisciplinary Intensive Care Research Organisation, St James’s Hospital, Dublin, Ireland; 22 Trinity College Dublin, Dublin, Ireland; 23 Centro de Investigacion Biomedica En Red Entermedades Respiratorias, Institute of Health Carlos III, Madrid, Spain; 24 Pulmonary Department, Hospital Clinic, Universitat de Barcelona, Barcelona, Spain; 25 Department of Surgery, Harborview Medical Centre, University of Washington, Seattle, USA; 26 Pandit Bhagwat Dayal Sharma Postgraduate Institute of Medical Sciences, Rohtak, India; 27 Section of Infectious Diseases, Department of Internal Medicine, Wake Forest University School of Medicine, Winston-Salem, NC USA; 28 Emergency Department, Policlinico Umberto I, Sapienza University, Rome, Italy; 29 Anesthesiology and Critical Care Medicine Department, DMU PARABOL, Bichat Hospital, AP-HP, Université Paris Cité, Paris, France; 30 Division of Trauma and Acute Care Surgery, Department of Surgery, Fundación Valle del Lili, Cali, Colombia; 31 Department of Surgical Science, University of Cagliari, Cagliari, Italy; 32 Global Health eCore, Vall d’Hebron University Hospital Campus, Barcelona, 08035 Spain; 33 Medicine Department, Universitat Internacional de Catalunya, Sant Cugat del Valles, Spain; 34 Department of Surgery, Western Michigan University Homer Stryker M.D. School of Medicine, Kalamazoo, MI USA; 35 Emergency Surgery and Trauma, Fondazione Policlinico Universitario A. Gemelli IRCCS, Rome, Italy; 36 Infectious Disease and Intensive Care Unit, Pontchaillou University Hospital, Rennes, France; 37 Department of Medicine, MetroHealth Medical Center, Cleveland, OH USA; 38 Division of Infectious Diseases and Geographic Medicine, University of Texas Southwestern Medical Center, Dallas, TX USA; 39 Emergency Surgery department, Meilahti Tower Hospital, HUS Helsinki University Hospital, Haartmaninkatu 4, Helsinki, Finland; 40 First Department of Surgery, Department of Abdominal, Thoracic Surgery and Traumatology, First Faculty of Medicine, Charles University and General University Hospital, Prague, Czech Republic; 41 Emergency and General Surgery Department, Bufalini Hospital, Cesena, Italy; 42 Department of Medicine, Division of Infectious Diseases, Northeast Ohio Medical University, Rootstown, OH USA; 43 General, Emergency and Trauma Surgery Unit, Pisa University Hospital, Pisa, Italy

**Keywords:** Antimicrobial resistance, Antimicrobial therapy, Intra-abdominal infections, Source control

## Abstract

Intra-abdominal infections (IAIs) are an important cause of morbidity and mortality in hospital settings worldwide. The cornerstones of IAI management include rapid, accurate diagnostics; timely, adequate source control; appropriate, short-duration antimicrobial therapy administered according to the principles of pharmacokinetics/pharmacodynamics and antimicrobial stewardship; and hemodynamic and organ functional support with intravenous fluid and adjunctive vasopressor agents for critical illness (sepsis/organ dysfunction or septic shock after correction of hypovolemia). In patients with IAIs, a personalized approach is crucial to optimize outcomes and should be based on multiple aspects that require careful clinical assessment. The anatomic extent of infection, the presumed pathogens involved and risk factors for antimicrobial resistance, the origin and extent of the infection, the patient’s clinical condition, and the host’s immune status should be assessed continuously to optimize the management of patients with complicated IAIs.

## Introduction

Intra-abdominal infections (IAIs) are a major cause of morbidity and mortality in hospital settings worldwide. However, data from most published clinical trials underestimate the true mortality risk for several reasons. First, patients with complicated acute appendicitis are often over-represented in clinical trials. Second, because clinical trials of investigational antibiotics for complicated IAIs (cIAIs) must demonstrate safety as well as efficacy, enrolled patients with IAIs have a low likelihood of mortality, due to specific trial eligibility criteria that exclude patients with shock or critical illness and sometimes, specific comorbidities. For example, registrational trials usually report a mortality rate of *≤* 2% [1], whereas mortality may exceed 20% from IAIs in critically ill patients [2], usually due to multiple organ dysfunction syndrome. The prospective observational Complicated Intra-Abdominal Infections Score Study (WISS), which included 4,553 patients with cIAIs from 132 medical institutions worldwide, showed in 2015 an overall mortality rate of 9.2% [3], which is likely a more accurate assessment of the overall mortality risk from IAIs.

Peritonitis refers to inflammation of the peritoneum. Infectious peritonitis can be categorized into primary, secondary, or tertiary peritonitis based on causation and anatomy. Primary (formerly “spontaneous”) bacterial peritonitis is a diffuse infection caused by a single bacterial species. There is no disruption of the gastrointestinal (GI) tract. Primary peritonitis typically occurs in patients with cirrhosis and ascites or patients with rheumatologic or chronic kidney disease (e.g., systemic lupus erythematosus). Patients with peritonitis associated with a peritoneal dialysis catheter, ventriculo-peritoneal shunt, or other device have primary peritonitis strictly speaking, but such cases are best considered as their own sub-category, device-associated peritonitis (DAP). Primary peritonitis is infrequent on surgical wards, but must be recognized because the treatment is medical and surgical intervention is not indicated. By contrast, DAP is usually treated by removing the device, especially for fungal or pseudomonal DAP (by surgery if necessary), and antibiotics [4]. Primary peritonitis and DAP caused by anaerobes are exceedingly unusual, so isolation of an anaerobe from presumed primary peritonitis or DAP requires evaluation for perforated viscus, which can be caused by erosion of the device.

Secondary peritonitis is the most frequent form of peritonitis presenting to surgical units. It results from the loss of integrity of the GI tract (e.g., GI necrosis, perforated viscus). It is invariably polymicrobial, but varies widely in severity and outcomes. The treatment involves surgical intervention, whether percutaneous or via laparoscopy or open procedure. Percutaneous drainage can obviate the need for surgery, or temporize until treatment confers better surgical risk, whereas an incision provides opportunity to achieve “source control”, which is the principal determinant of outcome. A short course of antibiotics is usually necessary, but adjunctive.

Tertiary peritonitis is a recurrent or persistent infection of the peritoneal cavity after more than one unsuccessful source control procedure for secondary peritonitis. Tertiary peritonitis is a distinct entity, representing an e complication of failed source control for secondary peritonitis [4]. The terms “ongoing peritonitis” or “persistent peritonitis” have been proposed as alternatives because they may describe better the evolution of secondary peritonitis. Patients with tertiary peritonitis are usually critically ill and require substantial hospital resources for nutritional and organ functional support, as well as re-laparotomy (sometimes multiple operations). The microbiology differs from secondary peritonitis in that is associated with commensal organisms (e.g., enterococci, yeast, *Staphylococcus epidermidis*, or *Pseudomonas aeruginosa*).

The term “acute peritonitis” is vague and is often used generically. cIAI nomenclature, as opposed to the term “uncomplicated intra-abdominal infections” (uIAIs) defines an infection proceeding beyond the organ of origin into the peritoneal cavity. cIAIs are complex conditions, in which numerous aspects of the disease process and management options must be assessed to provide optimal management [4]. The cornerstones of cIAI management include adequate source control achieved by the index procedure; appropriate antimicrobial therapy considering the host, the disease, the likely pathogen(s), and the agents available; and hemodynamic support by intravenous fluid, with judicious vasopressor use for refractory septic shock not responsive to fluid alone [4].

## Methods

This evidence-based position statement has been signed by a representative multidisciplinary working group of experts, with its main objective being to describe best practices for cIAI management. The group, representing the Global Alliance for Infections in Surgery, included general and emergency surgeons, intensive care specialists, and specialists in infectious diseases. The statement has been drafted by all contributors, following a comprehensive literature review of pertinent current scientific evidence. The supporting documentation was identified by a search conducted through PubMed (National Institutes of Health, Bethesda, MD, https://pubmed.ncbi.nlm.nih.gov) and Google Scholar (Alphabet, Inc., Mountain View, CA, https://scholar.google.com). The search identified articles published in English between January 2010 and December 2023, which were supplemented selectively by earlier articles for historical context or to emphasize timeless points. An expert reviewed the selected articles, and drafted the initial manuscript, which was shared with the experts’ group to construct a position statement about the management of cIAIs. The statements have been formulated and graded according to the Grading of Recommendations Assessment, Development, and Evaluation (GRADE) hierarchy of evidence. Evidence quality has been graded high, moderate, low, or very low according to the GRADE methodology. The strength of the recommendations has been classified as weak or strong.

For each statement, consensus among the experts was reached using a Delphi approach. Statements were endorsed as a strong recommendation with agreement by ≥ 80% of participating experts. The final document was approved by each working group member to ensure consensus. Concepts and approaches described are recommended plans of care, based on best available evidence and the consensus of experts, but they do not exclude other approaches as being within the standard of care.

### Recommendations for the management of complicated intra-abdominal infections

Determining the origin of the infection is crucial to defining the operative plan for source control. Factors of importance in the management of cIAIS are the following: (1) origin of the infection; (2) anatomic extent of infection; (3) presumed pathogens involved and risk factors for AMR; (4) clinical conditions; and (5) host immune status.

### Statement 1

**In patients with cIAIs, a personalized approach is crucial to optimize outcomes and should be based on multiple aspects requiring careful clinical assessment: (1) The anatomic extent of infection; (2) the presumed pathogens and risk factors for antimicrobial resistance (AMR); (3) the origin of the infection; (4) the patient’s clinical condition; and (5) the host’s immune status. Assessment should be continuous to optimize management (Very low-quality evidence, Strong recommendation).**

#### Origin of the infection

The term “intra-abdominal infections” includes several different pathologic conditions ranging in severity from uncomplicated appendicitis to diffuse fecal peritonitis [5]. The WISS study [1] confirmed that acute appendicitis is the most frequent cause of IAIs; one-third of cases are complicated. Acute appendicitis is usually caused by obstruction of the appendiceal lumen by an appendicolith or some other mechanical cause. Appendiceal primary tumors such as carcinoid tumors or adenocarcinoma, intestinal parasites, or hypertrophied lymphatic tissue may also cause lumenal obstruction. Acute appendicitis is postulated to develop when the obstructed appendiceal lumen develops bacterial overgrowth that, combined with luminal hypertension from mucus secretion and resulting cessation of venous outflow, causes acute infection with gangrene or perforation, and abscess formation or diffuse peritonitis. The natural history of appendicitis has been described in three stages, from: (1) Normal; to (2) uncomplicated acute appendicitis; and (3) eventually, complicated appendicitis [6].

Acute cholecystitis is the second most common cause of IAI, but most cases are uIAI. Gallstone-associated cystic duct obstruction is responsible for 90-95% of cases of acute cholecystitis. Approximately 5-10% of patients with cholecystitis have acute acalculous cholecystitis (AAC) defined as ischemic inflammation of the gallbladder in the absence of gallstones, typically in the setting of critical illness [7]. Rarely, AAC has a viral pathogenesis (e.g., cytomegalovirus, Epstein-Barr virus). Cystic duct obstruction precipitates prostaglandin-mediated gallbladder wall inflammation and edema. Early in the course of disease, one-half or more of acute cholecystitis cases are culture-negative, with bacterial invasion being a secondary phenomenon. The most common pathogens are *Escherichia coli*, *Klebsiella* spp., and *Enterococcus* spp. If untreated, mural ischemia leads to gangrene, although perforation (complicated acute cholecystitis) is uncommon. A gangrenous gallbladder may become infected by gas-forming organisms (e.g., *Clostridium* spp.) causing acute emphysematous cholecystitis, which can have a fulminant course. Gallbladder perforation can quickly become life-threatening if not contained by local host defenses (subhepatic abscess); perforation with free rupture and generalized peritonitis has a high rate of mortality comparable to other causes of diffuse bacterial peritonitis.

Acute diverticulitis begins with inspissation and inflammation of colonic diverticuli, which may cause local ischemia and progress to colonic micro- or macro-perforation. It may be classified according to the extent of inflammation and related complications that it induces, ranging from mild uncomplicated episodes to major complications (e.g., abscess or peritonitis). In Western societies, most diverticula are found in the sigmoid and left colon, whereas diverticula of the right colon are more common in Asian populations [5].

Gastroduodenal ulcer perforations have decreased recently, owing to the widespread prescribing of proton pump inhibitors (and over-the-counter availability), a decreasing incidence of *Helicobacter pylori* infection in Western countries, and effective multi-drug therapy when diagnosed in the non-acute setting [5]. However, perforated gastroduodenal ulcer disease is still a common emergency condition worldwide and is associated with high mortality if not treated promptly. The main causal factors include non-steroidal anti-inflammatory drugs, systemic corticosteroids, tobacco smoking, *H. pylori* infection, and high-sodium diets, all of which are associated with gastric acid hypersecretion. The above-mentioned medications also disrupt the gastric mucus barrier and mucosal H^+^ efflux pumps. Most community-onset gastroduodenal perforations are sterile if source control is achieved within the first 24 h after perforation, with antibacterial therapy for invasive infection (as opposed to prophylaxis) only necessary thereafter. The most common microbial isolate after gastroduodenal perforation is *Candida* spp., which is usually a contaminant from the esophago-gastric microbiome that does not require antifungal therapy. By contrast, stress-related gastric mucosal injury (stress “ulceration,”) now rare even among critically ill patients in intensive care units (ICUs), is caused by gastric ischemia/reperfusion injury. Characterized by diffuse erosive gastritis rather than discrete ulcers, stress gastritis is more likely to result in upper GI hemorrhage than gangrene or perforation.

Small bowel perforations are less common as causes of peritonitis in Western countries compared with other areas of the world. In Western countries, most small intestinal perforations are due to unrecognized intestinal ischemia (mesenteric or obstruction/strangulation) inflammatory bowel disease (e.g., Crohn disease) or occasionally from ingested foreign bodies or small intestinal diverticulitis (e.g., Meckel type). By contrast, in most of the world, small bowel perforations are usually due to typhoid fever (*Salmonella typhimurium*). Typhoid fever is endemic in Asia, Africa, Latin America, the Caribbean, and Oceania [5]. Typhoid ileal perforation is a major global public health problem because of high morbidity and a mortality rate up to 60% [8], and it can present literally anywhere worldwide owing to international travel.

Post-operative peritonitis (PP) is a life-threatening, hospital-acquired cIAI with high rates of mortality [5]. As it is rare compared with community-onset cIAI and patients with PP can become critically ill, the condition is often excluded from registrational trials of new antimicrobial agents, and thus under-studied. The most common cause of PP is anastomotic leak [5], which is most frequent after rectal resection, but it may complicate any GI anastomosis or suture line. Other causes include bile or urine leaks in the appropriate context, and failed source control from the index procedure. The diagnosis of PP may be challenging owing to a lack of specific confirmatory clinical signs. Atypical or unrecognized clinical presentations may result in hazardous diagnostic and therapeutic delays. Management of PP includes supportive therapy of organ dysfunction, source control of infection, antimicrobial therapy, and the possibility of open-abdomen management and complex, staged abdominal wall reconstruction.

Pelvic inflammatory disease (PID) is an infection of the upper part of the female genital tract, including the uterus, fallopian tubes, or adjacent pelvic structures causing peritonitis that may spread from the pelvis to the abdomen [5]. The cause is ascending bacterial infection from the vagina in 85% of cases, most commonly from sexually transmitted pathogens (*Neisseria gonorrhoeae*, *Chlamydia trachomatis*). Endogenous vaginal and cervical flora, especially anaerobes, cause PID in most of the remaining cases. Most cases are treated as outpatients with antibiotics alone. Indications for hospitalization include failure of outpatient management, severe illness, and pregnancy (owing to the risk of pregnancy loss). Pelvic (tubo-ovarian) abscess may be an indication for surgery.

Trauma, a major global public health problem, can be associated with high morbidity and mortality depending on mechanism, injury pattern, time to stabilization, and host frailty regardless of socioeconomic status [5]. Both blunt and penetrating mechanisms may result in bowel injury; motor vehicle crashes are the most common of blunt intestinal injury. followed by falls [5]. The small intestine is more likely than colon to be injured by either mechanism. Infection is considered established (as opposed to contamination by enteric contents) after 12 h for colon injury and 24 h for other locations; within those time frames only surgical antimicrobial prophylaxis (maximum 24 h; ideally a single dose) is required, even if the colon is lacerated. Blunt hollow viscus injury may have a more insidious presentation in this setting, possibly resulting in delayed diagnosis and intervention and consequent adverse outcomes.

### Statement 2

**IAI is the setting where source control is most impactful. Achievement of source control is the single most important determinant of patients’ outcomes from cIAI, allowing for short-course antibiotic therapy that reduces antibiotic selection pressure. Source control should be undertaken promptly to remove devitalized tissue and infected fluid and prevent ongoing contamination (Low-quality evidence, Strong recommendation).**

Source control encompasses all measures aiming to identify and eliminate the source of infection, and control the ongoing contamination [9].

Achievement of source control is of utmost importance in the management of cIAIs. In these settings, source control improves patient outcomes. Although not tested definitively by randomized controlled trials (RCTs) the magnitude of the increased (up to 10-fold) risk of death and other adverse outcomes associated with delayed or inadequate source control makes clear its primacy. Moreover, source control is associated with shorter-course antibiotic therapy [10]. Although appropriate source control is the standard of care for patients with cIAIs, some selected patients with uIAIs (e.g., mild appendicitis and diverticulitis) can be treated successfully with antibiotic therapy alone.

The goals of any source control procedure are to remove infected or devitalized tissues, opening of spaces and compartments to mitigate sequestration or persistent infection, evacuation of pus or other fluids, and irrigation (“washing out”) of the abdominal cavity. Selection of a specific source control procedure should be based for each patient on the characteristics of the infection and the patient, as well as the availability of technical expertise. Technical options for source control, alone or in combination with antimicrobial therapy (depending on the circumstance) include: (1) Resection or suture plication of a perforated viscus; (2) removal of an infected organ (e.g., appendix, gallbladder); (3) debridement of necrotic tissue or resection of infarcted bowel; (4) drainage of an abscess or infected peritoneal fluid; and (5) primary anastomosis or fecal diversion after bowel resection. Copious intraoperative peritoneal lavage is no longer recommended for localized infection. Several recent series and one prospective trial [11–13] of patients with perforated appendicitis found that aspiration and limited irrigation to remove gross contamination were as effective as lavage. More extensive lavage may be needed for diffuse peritonitis.

Source control can be also achieved sometimes by a less-invasive technical approach such as ultrasound (US)- or computed tomography (CT)-guided percutaneous drainage [4]. Percutaneous drainage is a safe and mostly (~ 85%) effective procedure that allows minimally invasive evacuation of abdominopelvic abscesses and fluid collections with lower morbidity and mortality when appropriate anatomically and feasible technically, depending on the preference of the performing radiologist or surgeon and the location of the abscess/fluid collection. Successful source control (curative drainage) is defined as complete, definitive resolution of infection by an index procedure that requires no further intervention. In most studies, source control is achieved in more than 80% of patients [14]. Partial success is defined as adequate temporizing drainage to stabilize the patient, with surgery performed subsequently to repair the underlying problem. Partial success rates vary, but generally range from 5 to 10% of patients [14]. In certain conditions, the expectation of drainage is to serve as a “bridge” until definitive surgical treatment can be performed. For the purposes of these statements, “bridge” therapy is considered partial success even though the treatment plan may include multiple, staged interventions. Failure occurs about equally because of persistent or recurrent collections. These results are similar for abdominal and chest percutaneous drainage [14].

### Statement 3

**The origin of the infection should be always investigated for treatment planning. In physiologically deranged patients (i.e., septic shock), early exploration may be performed even if the source of infection is not defined (Very low-quality evidence, Strong recommendation).**

The diagnosis of cIAI is based primarily on clinical and radiologic assessment. A typical patient is admitted to the emergency department with abdominal pain and a systemic inflammatory response, including two or more of fever, tachycardia, tachypnea, and leukocytosis or leukopenia (i.e., systemic inflammatory response syndrome). Abdominal tenderness and involuntary guarding suggest the presence of a cIAI. A complete blood count is the most common laboratory investigation, although it is insensitive and relatively non-specific in cIAI. Inflammatory biomarkers such as C-reactive protein and procalcitonin (PCT) have been evaluated [15, 16]. US and CT are essential diagnostic tools. US is portable and can be performed at the bedside by surgery or radiology. Impediments to US are ileus (increased bowel gas) and obesity, which may mask visualization. It is also strongly operator-dependent, with imaging and interpretation performed ideally by the same individual. US has been the preferred initial diagnostic modality for children and for acute cholecystitis of adults, but increasingly, and for most other indications within cIAI, the higher diagnostic accuracy of CT for identification of the source of infection has been established [17–20], especially for stable patients for whom CT with intravenous contrast is the imaging modality of choice. Intravenous contrast-enhanced CT provides superior anatomic detail [21]. CT not only supports the diagnosis but also informs (and facilitates, in the circumstance of percutaneous drainage) treatment [21]. A step-up strategy with CT performed after an inconclusive or negative US has been proposed as a safe alternative approach for patients with IAIs, especially in the setting of acute diverticulitis [22, 23]. Magnetic resonance imaging may be useful for diagnosis of the acute abdomen [24], but its routine application is limited by the challenges imposed by the emergency setting.

Rapid, accurate identification of the infection source is especially crucial in managing critically ill patients with cIAI. Delay in achieving surgical source control (i.e., > 6 h from sepsis onset) [25] portends worse outcomes. For critically ill, physiologically deranged patients (i.e., septic shock), early exploration may be recommended even if the source of infection remains unclear despite imaging [26].

#### Anatomic extent of infection

Assessing the origin and extent of the infectious process is important to define the treatment approach. In uIAIs, the infectious process only involves a single organ, not extending beyond. In cIAIs, the infectious process extends beyond the organ into the peritoneal cavity, leading to abscess formation or diffuse peritonitis [4]. A limitation of this classification is that it does not describe patients’ complexity. On the other hand, in its simplicity, extension of the infectious process identifies those patients who need both source control and antibiotic therapy. In principle, patients with uIAIs (e.g., most cases of acute appendicitis and cholecystitis) can be managed with either surgical source control or antibiotics alone [27–29]. Patients with uncomplicated appendicitis or acute cholecystitis undergoing adequate source control do not need post-operative antibiotics [30–32].

### Statement 4

**In the event of uIAIs, such as acute appendicitis or acute cholecystitis, if the focus of infection is controlled by appropriate surgical management, post-operative antibiotic therapy is not necessary. In the event of cIAIs, treatment generally involves source control and antibiotic therapy. Selected patients with perforated diverticulitis (including those with an abscess < 4 cm in diameter), a peri-appendiceal mass or phlegmon, or a perforated gastroduodenal ulcer (i.e., no sepsis, peritonitis, or extravasation by water-soluble contrast gastroduodenography) can be managed without definitive source control if responding satisfactorily to antibiotic therapy and other supportive measures (Low-quality evidence, Strong recommendation).**

### Statement 5

**For patients with uncomplicated acute appendicitis, appendectomy is the standard treatment. If source control is adequate, post-operative antibiotic therapy is not necessary. Treatment by antibiotics alone may be used in selected non-pregnant patients with uncomplicated acute appendicitis and no appendicolith. However, non-operative management (NOM) is less effective than surgery in the long term due to a high recurrence rate (High-quality evidence, Strong recommendation).**

### Statement 6

**In patients with uncomplicated acute cholecystitis, early laparoscopic cholecystectomy is the standard treatment. If source control is adequate, post-operative antibiotic therapy is not necessary. If early cholecystectomy is not performed, interval cholecystectomy should be planned between 6 and 12 weeks after the episode of acute cholecystitis (Moderate-quality evidence, Strong recommendation).**

### Statement 7

**In immunocompetent patients with uncomplicated mild acute diverticulitis, antibiotic therapy may be omitted (Moderate-quality evidence, Weak recommendation).**

Although the “gold standard” treatment of acute appendicitis is appendectomy [33], antibiotic therapy alone has been proposed for uncomplicated acute appendicitis of non-pregnant patients without an appendicolith. However, NOM of acute appendicitis, which requires CT confirmation, is demonstrably less effective in the long term due to 1-year recurrence rates of up to 40% [30–32].

For therapy of acute uncomplicated cholecystitis, two options have been proposed. The early option includes laparoscopic or open cholecystectomy within a few days of symptom onset, providing immediate, definitive treatment in the same hospital admission. The delayed option includes antibiotic therapy, possibly with a temporizing percutaneous cholecystostomy, followed by cholecystectomy after an interval of 6–12 weeks, during which time the acute inflammation subsides [4]. Early laparoscopic cholecystectomy is the treatment of choice for acute cholecystitis, due to shorter hospitalization and no increased morbidity compared with delayed cholecystectomy. However, RCTs and meta-analysis reported a wide array of timing in performing early cholecystectomy for acute cholecystitis, up to 96 h from admission or up to 1 week from symptom onset. Lyu et al. [34], who included 15 RCTs (1,669 patients) in a systematic review and meta-analysis, found that early laparoscopic cholecystectomy was comparably safe and effective compared with delayed cholecystectomy when performed within 7 days of presentation. No differences were found in terms of bile duct injury, surgical site infection, total complications, or conversion to open surgery. Early cholecystectomy was associated with a significantly shorter duration of hospital stay, due entirely to minimizing preoperative delay.

For some cases of uncomplicated acute diverticulitis, NOM without antibiotics is effective [35–38]. A 2017 RCT (DIABOLO trial) of observation *versus* systemic antibiotic treatment [37] for a first episode of CT-proved acute left-sided colonic diverticulitis of Hinchey grades 1a and 1b included 528 patients. Median time to recovery was 2 days longer for observation (hazard ratio [HR] for recovery of 0.91, *p* = 0.151), but hospital stay was significantly shorter. No differences were found for secondary endpoints of complicated, ongoing, or recurrent diverticulitis; sigmoid resection; readmission; adverse events, or mortality. Outcomes of DIABOLO study enrollees at 24 months’ follow-up [39] showed no differences in rates of recurrent or complicated diverticulitis, or sigmoid resection.

Approximately 15–20% of patients admitted with acute diverticulitis have an abscess by CT scan [40]. Smaller diverticular abscesses may be treated by systemic antibiotics alone with or without percutaneous drainage, depending on the diameter of the abscess, with up to ~ 4 cm accepted as a reasonable limit for treatment with antibiotic therapy alone [40]. Although the prevalence of perforated diverticulitis complicated by generalized peritonitis (Hinchey 3–4) is low, there is a risk of mortality regardless of the surgical approach. However, distant free air by CT (a known predictor of failure of NOM) does not obligate surgical intervention. Dharmarajan et al. [41] described successful NOM for patients with acute diverticulitis and pneumoperitoneum, excluding those with hemodynamic instability. Sallinen et al. [42] reported results of NOM in patients with CT-verified extra-luminal gas. NOM was feasible for hemodynamically stable patients with pericolic extra-luminal gas, or only a “small amount” of distant intraperitoneal gas in the absence of clinical diffuse peritonitis or fluid in the fossa of Douglas. However, a large amount of distant intra- or retroperitoneal gas was associated with a high failure rate (~ 60%) of NOM, even in the absence of generalized peritonitis.

Antibiotics alone may be used to treat selected patients with uncomplicated acute appendicitis, although there is a significant risk (~ 40%) of subsequent recurrence [43]. In patients with complicated appendicitis presenting with abscess or phlegmon, optimal management is controversial. One study showed that NOM for a peri-appendiceal abscess or mass resulted in fewer complications and shorter hospitalization [43].

### Statement 8

**In patients with cIAIs undergoing adequate source control, 4 days of fixed-duration antibiotic therapy is sufficient. In the setting of complicated acute appendicitis, the duration of antibiotic therapy may be shortened further in selected patients (High-quality evidence, Strong recommendation).**

### Statement 9

**Ongoing signs of abdominal infection or systemic illness after 7 days of antibiotic therapy warrant cessation of therapy and a diagnostic investigation rather than prolongation or modification of antibiotic therapy (Low-quality evidence, Strong recommendation).**

### Statement 10

**Biomarkers such as procalcitonin can guide antibiotic duration in patients with signs of ongoing infection (Low-quality evidence, Weak recommendation).**

Short-duration antimicrobial treatment of cIAIs improves outcomes while adhering to the principles of antimicrobial stewardship [44]. The prospective STOP-IT trial [45] demonstrated that, for patients with cIAIs undergoing adequate source control, outcomes after ~ 4 days of fixed-duration antibiotic therapy were similar to outcomes after a longer course (~ 8 days) of antibiotics that extended until 48 h after the resolution of physiochemical abnormalities. STOP-IT trial data were also evaluated retrospectively to identify risk factors identified with treatment failure [46], which included corticosteroid use, Acute Physiology and Chronic Health Evaluation (APACHE)-II score ≥ 5 points, hospital-acquired infection, or a colon source of cIAI. Despite the presence of risk factors, there were no differences in treatment failure rates between groups, indicating that even patients at high risk of treatment failure do not benefit from longer-duration therapy.

The course of antibiotic therapy may possibly be shortened in the setting of acute appendicitis. A recent open-label, non-inferiority trial of patients with complicated appendicitis (aged ≥ 8 years) showed that 2 days of postoperative intravenous antibiotics was non-inferior to 5 days of therapy in terms of infectious complications and 90-day mortality [47]. However, a majority of enrolled patients had gangrenous (non-perforated) appendicitis that requires only 24 h of antimicrobial therapy. Given the paucity of data regarding optimal duration of therapy for critically ill patients with cIAI, there may be considerable practice variation. Critically ill surgical patients often receive antibiotics longer than necessary. Antibiotic therapy may be truncated in these patients also, as demonstrated by the DURAPOP RCT of critically ill patients with postoperative IAIs [48].

Patients who have ongoing signs of infection after a source control procedure and appropriate fixed-duration antibiotic treatment need investigation to assess for an ongoing source of infection (failed source control *versus* untreated resistant pathogen[s]) to determine whether re-operation is necessary, rather than prolongation or modification of the antibiotic treatment regimen. PCT may be useful for individualizing antibiotic use. Clinical PCT-based algorithms are used as part of antibiotic stewardship programs in various settings. Serum PCT concentration in healthy unoperated individuals is typically < 0.05 mcg/L, but may be higher postoperatively due to a PCT response to tissue injury. In the presence of bacterial infection, higher PCT concentrations correlate with severity of bacterial infection, whereas a decreasing concentration usually reflects improvement and resolution of infection.

Evidence shows that PCT monitoring can reduce antibiotic duration safely in critically ill patients [49–53]. Furthermore, PCT can inform the duration of antibiotic therapy in cIAIs [54–56], and can be particularly useful for patients with ongoing inflammation, in that decreasing concentrations in the postoperative period can guide the cessation of therapy. Three studies show that a PCT-based algorithm may decrease antibiotic exposure in patients with cIAIs. Huang et al. [54] conducted a prospective study to investigate whether a PCT algorithm could reduce antibiotic therapy duration in patients with cIAIs undergoing surgery. PCT concentrations were evaluated pre-operatively, serially for the first week post-operatively, and subsequently if needed. Antibiotic therapy was discontinued if [PCT] was < 1.0 ng/L or decreased by 80% *versus* day 1 with resolution of clinical signs. The PCT algorithm significantly reduced antibiotic therapy duration (PCT group, 3.4 days *versus* 6.1 days in the control group). Maseda et al. [55] published a retrospective study of 121 consecutive critically ill surgical patients (ICU duration > 48 h) treated for cIAI. Treatment duration was reduced by 50% in the PCT group regardless of hemodynamics. Slieker et al. [56] investigated whether PCT could tailor postoperative antibiotic therapy in surgical patients with cIAIs. In a subgroup of patients with GI perforation, duration of antibiotic therapy was 3 days shorter (7 d *versus* 10 d) in the PCT group.

#### Individual patient risk for presumed pathogens involved and risk factors for antimicrobial resistance

Accurate patient stratification is crucial to optimize empiric antibiotic therapy in this era of AMR [57].

### Statement 11

**Narrow-spectrum antibiotic regimens having activity against typical gram-negative*Enterobacterales***, **gram-positive cocci, and obligate anaerobes should be used to treat patients with community-acquired cIAIs. In this setting, broad-spectrum or additional agents to provide anti-pseudomonal or anti-enterococcal coverage or antifungal therapy should not be used routinely (Low-quality evidence, Strong recommendation).**

### Statement 12

**For patients with hospital-acquired IAIs, agent(s) with broad-spectrum activity are preferred (Moderate-quality evidence, Strong recommendation).**

### Statement 13

**Patients who have received recent broad-spectrum antimicrobial therapy, had prolonged hospitalizations, underwent multiple invasive interventions, or are known to have been colonized or infected with a resistant gram-negative organism should be considered at risk for infection from a resistant gram-negative pathogen (Moderate-quality evidence, Strong recommendation).**

### Statement 14

**Antibiotic regimens with activity against multidrug-resistant (MDR) bacteria should be used for selected patients with IAIs who are strongly suspected or proved to be harbouring a resistant pathogen (Moderate-quality evidence, Strong recommendation).**

Initial antibiotic therapy for cIAIs is typically empiric in nature because standard microbiologic data and susceptibility results generally require 24–72 h after peritoneal fluid specimen collection. Blood cultures are seldom positive in cIAI (< 10%) and therefore largely unreliable. Typical bacterial pathogens in IAIs reflect endogenous gut flora including *Enterobacterales* such as *E. coli* and *Klebsiella* spp., viridans group *Streptococcus*, and anaerobes (especially *Bacteroides* spp.) [4]. Historically, cIAI pathogens tended to have greater susceptibility in community- as opposed to hospital-acquired infections.

IAIs caused by susceptible bacteria may be managed by beta-lactam/beta-lactamase inhibitor combinations (e.g., ticarcillin/clavulanic acid or piperacillin/tazobactam), or a non-pseudomonal carbapenem (e.g., ertapenem) [4]. Increasing AMR to amoxicillin/clavulanic acid among *E. coli* and other *Enterobacterales* worldwide has compromised its clinical utility for empiric therapy; it should only be prescribed as targeted therapy based on demonstrated susceptibility. The comparably broad-spectrum activity of piperacillin/tazobactam makes it still useful in managing severe IAIs. However, the anti-pseudomonal activity of piperacillin/tazobactam is unnecessary for most community-acquired cIAIs, and its use in hospital-acquired infections should be determined based on local microbiologic epidemiology [58].

Many isolates of *E. coli* and other *Enterobacterales* are susceptible to third-generation cephalosporins that, in combination with metronidazole, may be options for empiric therapy of non-severe IAIs. Cefepime is a fourth-generation cephalosporin with a broader spectrum of activity than third-generation cephalosporins. Compared to ceftriaxone, cefepime is poorly hydrolysed by AmpC beta-lactamase, allowing it to be effective against AmpC-producing organisms. For empiric therapy, cefepime must also be given with metronidazole [59]. Fluoroquinolones have been prescribed widely for the treatment of IAIs because of their putative activity against aerobic gram-negative bacteria, tissue penetration, and high oral bioavailability. However, resistance of *E. coli* and *Klebsiella* spp. to fluoroquinolones has increased substantially over time [4, 59], limiting their use for empiric treatment of IAIs.

In a 2019 multinational observational cohort study of IAIs in 2,621 ICU patients [60], infection was community-acquired in 31.6% of patients and hospital-acquired in 68.4%. Culture and susceptibility testing were performed for 1,982 (75.6%) of patients. Gram-negative organisms were the most frequent bacteria isolated (58.6%). *Enterobacterales* was the most common bacterial order (51.7%) and *E. coli* was the pathogen isolated most frequently (36.8%). Gram-positive aerobic bacteria were isolated from 39.4% of patients. AMR was common (26.3%), without significant differences between community- and hospital-acquired IAIs.

In 2012, the European Centre for Disease Prevention and Control (ECDC) and the U.S. Centers for Disease Control and Prevention (CDC) developed standardized nomenclature to describe acquired resistance profiles in bacteria [61]. MDR was defined as acquired non-susceptibility to at least one antibiotic in three or more antibiotic classes (e.g., cephalosporins, fluoroquinolones, tetracyclines). Extensively drug-resistant (XDR) bacteria were defined as non-susceptible to at least one antibiotic in all but two or fewer antibiotic classes (bacterial isolates remain susceptible to only one or two classes). Pan-drug-resistant (PDR) bacteria were defined as non-susceptible to all antibiotics in all antibiotic classes.

In the past, predicting potential resistance patterns was based on establishing whether the infection was community- or hospital-acquired. Given the increasing rate of extended-spectrum beta-lactamase (ESBL)-producing *Enterobacterales* and carbapenem-resistant *Enterobacterales* (CRE) observed in community-acquired infections [59], the choice of empiric antibiotic therapy has been complexified. Factors that should be considered to identify risk for resistant bacteria include previous colonization with an MDR pathogen, recent exposure to antibiotics or invasive procedures, and comorbidities or poor functional status [62]. Also, international travel affects the gut microbiota and is considered a risk factor for the acquisition of MDR pathogens [63]. Screening for carriage of CREs is an important global infection prevention and control tactic in endemic regions [64].

In the context of IAIs, the most common resistance phenotype is Extended-Spectrum Beta-Lactamases (ESBLs), which are widely prevalent in nosocomial infections and increasingly so in community-acquired infections [59, 65]. Among patients with risk factors for ESBL-producing *Enterobacterales*, especially if unstable, empiric antibiotics should have an anti-ESBL spectrum. However, among non-critically ill patients, a survival benefit from empiric antibiotic therapy has not been demonstrated consistently, and empiric anti-ESBL coverage may be unnecessary [66]. Carbapenems have been considered the antibiotics of choice to treat ESBL-producing *Enterobacterales*. Group 1 carbapenems include ertapenem, a once daily-carbapenem sharing the same activity of Group 2 carbapenems against ESBL-producing *Enterobacterales* [67] and anaerobes. Group 2 carbapenems include imipenem-cilastatin, meropenem, and doripenem. Compared to ertapenem, Group 2 agents have activity against *P. aeruginosa*. Unlike meropenem and doripenem, imipenem-cilastatin is active against ampicillin-susceptible enterococci.

The role of piperacillin/tazobactam in treating patients with ESBL-producing *Enterobacterales* has been debated [59]. Gram-negative bacteria may express multiple ESBLs as well as AmpC beta-lactamases concomitantly, and can manifest other mechanisms of resistance, limiting the activity of piperacillin/tazobactam [63]. On the other hand, the activity of beta-lactam agents, including piperacillin/tazobactam, is influenced by the “inoculum effect”, an increase in the minimum inhibitory concentration (MIC) of an antibiotic when the inoculum size is larger [63]. A RCT conducted in patients with ESBL-producing *Enterobacterales* blood stream infections showed inferior results of piperacillin/tazobactam compared to carbapenems [68]. Although piperacillin/tazobactam is not considered the first-choice antibiotic to treat ESBL-producing *Enterobacterales* [69], it may be an option for IAIs with adequate source control when bacteria are susceptible (MIC ≤ 4 mg/L) [59]. A high dose or prolonged/continuous infusion should be prescribed to optimise pharmacokinetics (PK) targeting in critically ill patients [70].

Aminoglycosides have in vitro activity against aerobic gram-negative bacteria, including ESBL-producing *Enterobacterales*, and can act synergistically with beta-lactam agents against certain gram-positive bacteria. Because of their toxicity, including nephro- and oto-toxicity, aminoglycosides are not recommended for the empiric treatment of IAIs [59]. They are generally prescribed for patients with beta-lactam allergies or used for synergy for brief periods in combination with beta-lactam agents against difficult-to-treat pathogens.

Tigecycline remains a limited option for treating patients with MDR cIAIs, due to its favorable coverage against anaerobic organisms, enterococci (including vancomycin non-susceptible strains), and ESBLs [71]. It is not active against *P. aeruginosa* or certain unusual IAI pathogens including *Proteus* spp. and *Serratia* spp. Excess mortality was observed in 12/13 phase 3 and 4 clinical trials of tigecycline, especially to treat bacteremic ventilator-associated pneumonia [72]. Tigecycline should be considered only when other therapeutic alternatives do not exist, and are contraindicated for treating hospital-acquired pneumonia or bacteremia. Eravacycline, a broad-spectrum fluorocycline, is structurally similar and demonstrates comparable broad-spectrum activity, including inactivity against *P. aeruginosa* [73], but a better safety profile. Eravacycline was investigated for cIAI treatment by 2 RCTs, in which it was non-inferior at test-of-cure (TOC) compared with 2 carbapenems (IGNITE 1: 87.0% for eravacycline *versus* 88.8% for ertapenem; IGNITE 4 90.8% *versus* 91.2% for meropenem) [74, 75]. Also, a low risk of *Clostridioides difficile* infection was observed after eravacycline treatment [76].

Fosfomycin is a broad-spectrum antibiotic with a wide therapeutic range and excellent tissue penetration [77]. Fosfomycin is administered frequently in Europe, in combination with other antibiotics, to combat severe bacterial infections, but seldom in North America. Monotherapy with Fosfomycin is not recommended owing to the rapid emergence of resistance.

Both ceftolozane/tazobactam and ceftazidime/avibactam have appropriate activity to treat cIAIs caused by ESBL-producing *Enterobacterales* [78, 79]. These agents evade carbapenemases that hydrolyze penicillins, cephalosporins, first-generation beta-lactamase inhibitors, and carbapenems. New agents for CRE, especially *K. pneumoniae*, include meropenem/vaborbactam and imipenem-cilastatin/relebactam [80, 81]. CRE usually possesses multiple potential mechanisms of antibiotic resistance [82]. Meropenem-vaborbactam and imipenem-cilastatin/relebactam are active against most *Enterobacterales* producing *K. pneumoniae* carbapenemases (KPC) but not those producing OXA-48-like carbapenemases.

Metallo-beta-lactamases (MBLs) are unique because they are zinc- dependent enzymes. MBLs hydrolyze most beta-lactam agents, including carbapenems, except for aztreonam. Ceftazidime/avibactam plus aztreonam, or cefiderocol are treatment options for MBL-producing *Enterobacterales*, although the latter has not been studied in cIAI. Cefiderocol is a novel cephalosporin that overcomes three distinct mechanisms of carbapenem resistance-avoiding porin channels and efflux pumps by entering bacterial cells via iron transport mechanisms-and stability to all four classes of beta-lactamases [83]. The new agent sulbactam/durlobactam and the investigational combinations of aztreonam/avibactam [76] and cefepime/taniboribactam show promise against MDR gram-negative bacilli, but none have been studied in cIAI.

Alarming rates of resistance have been described for non-fermenting gram-negative bacteria, including *P. aeruginosa*, *Stenotrophomonas maltophilia*, and *Acinetobacter baumanniii* complex. These difficult-to-treat MDR bacteria are intrinsically resistant to many antibiotics, but also can acquire resistance to most classes of antibiotic agents. Multiple mechanisms may be present simultaneously, conferring resistance to several classes of antibiotics [63], including membrane permeability defects, expression of efflux pumps, and production of antibiotic-hydrolysing enzymes such as AmpC beta-lactamases or carbapenemases. *S. maltophilia* and *A. baumanniii* complex are rare cIAI pathogens, but can cause postoperative pneumonia among high-risk critically ill patients with cIAI.

Among gram-positive bacteria, *Enterococcus* spp. is associated with increased morbidity in IAIs, but the effect on mortality is uncertain [84–86]. Whereas the role of enterococci in high-risk patients is well documented, their role in cIAIs in low-risk patients is doubtful [87]. Zhang et al. found by meta-analysis that anti-enterococcal regimens provide no improvement in cIAI treatment success, with similar mortality and adverse effects, in RCTs enrolling young patients with lower-risk community-acquired infections (median APACHE-II score, 6 points) [87]. Malignant disease, corticosteroid use, surgery, any antibiotic treatment, admission to an ICU, and an indwelling urinary catheter each predisposed patients with cIAI to a higher risk of enterococcal infection. The prevalence of enterococcal isolation was 2- to 5-fold higher from hospital-acquired IAIs.

Empiric anti-enterococcal therapy for cIAI is not necessary for most community-acquired infections, but is indicated in hospital-acquired IAIs and may be considered for immunocompromised patients, critically ill patients with sepsis and previous antimicrobial therapy lacking enterococcal coverage, or patients with valvular heart disease or intravascular prosthetics at high risk for endocarditis or blood-borne device-associated infection. The ideal regimen for high-risk patients is undetermined. *E. faecalis* is generally susceptible to ampicillin whereas *E. faecium*, encountered increasingly, is almost always ampicillin-resistant [46] and 70% of strains are resistant to vancomycin. The first-line treatment of glycopeptide-susceptible *E. faecium* is vancomycin. Linezolid or daptomycin may be used to treat vancomycin-resistant *E. faecium*, but occasional resistance has been reported to both agents. The glyclcyclines also have useful activity against vancomycin-resistant *E. faecium*.

### Statement 15

**In patients with cIAIs at risk of resistant pathogens, culture and susceptibility testing of peritoneal fluid at the site of infection should always be obtained. If resources are available, cultures should be obtained from all patients with cIAIs to analyze epidemiologic data that can be used to guide empiric antibiotic therapy (Low-quality evidence, Weak recommendation).**

Obtaining cultures from fluid allows escalation of an antimicrobial regimen if the initial choice is too narrow, and conversely de-escalation or discontinuation if the empiric regimen is too broad. When a microorganism is identified in culture, antimicrobial susceptibility testing should always be performed and reported to guide antibiotic therapy. Reporting is not without controversy, as selective non-disclosure (cascading) of antimicrobial susceptibilities is sometimes undertaken by antimicrobial stewardship programs to influence prescribing.

Although susceptibility testing has little impact on the treatment of community-acquired cIAIs such as appendicitis [88, 89], MDR bacteria can also cause community-acquired infections. In patients with cIAIs at risk of resistant pathogens, cultures of peritoneal fluid from the site of infection should always be obtained. Furthermore, susceptibility tests promote knowledge of local microbiologic epidemiology. Therefore, cultures should be obtained in all patients with cIAIs if resources are available.

Because early, targeted antibiotic therapy is crucial for improving patient outcomes especially in critically ill patients, prompt availability of diagnostic test results is a central theme in policy initiatives to combat infections, especially in sepsis [90].

### Statement 16

**For adults with sepsis (multiple organ dysfunction syndrome) or shock due to hospital-acquired cIAIs, empiric antifungal therapy for** ***Candida*** **spp. should be considered, especially those with recent abdominal surgery or gastrointestinal anastomotic leak. Empiric antifungal therapy may be considered for high-risk patients with community-acquired infections (Low-quality evidence, Strong recommendation).**

Intra-abdominal candidiasis is a rare but serious infection with a high mortality rate, especially of critically ill patients. The most common risk factors are GI perforation, anastomotic leak, and previous exposure to antifungal or antibacterial agents [91]. The challenge of intra-abdominal candidiasis is related partly to the diagnostic difficulty of differentiating between contamination and infection when *Candida* spp. is isolated. Invasive intra-abdominal candidiasis (as opposed to contamination) requires aggressive antifungal treatment, even though adequate source control is the most important factor in improving outcomes of these patients [92]. Unfortunately, early diagnosis of intra-abdominal candidiasis remains a challenge. Numerous risk factors for intra-abdominal candidiasis have been identified. Some clinical prediction rules were developed and validated to identify ICU patients at high risk of intra-abdominal candidiasis. In 2006, a Spanish group, using the database of the Estudio de Prevalencia de CANdidiasis project, proposed the “Candida score” to identify patients with high risk of intra-abdominal candidiasis; this score was calculated as 1 × (total parenteral nutrition) + 1 × (surgery) + 1 × (multifocal Candida colonization) + 2 × (severe sepsis) [93].

Empiric antifungal therapy for *Candida* spp. is typically not recommended for patients with IAIs, with the exceptions of immunocompromised patients, those with multiple risk factors, and critically ill patients. The guidelines of the European Society of Intensive Care Medicine (ESICM) and the Critically Ill Patients Study Group of the European Society of Clinical Microbiology and Infectious Diseases (ESCMID) suggest empiric antifungal therapy in patients with septic shock and multiple organ dysfunction syndrome [94]. By contrast, the guidelines of the Infectious Diseases Society of America (IDSA) suggest empiric antifungal therapy for patients with clinical evidence of IAI and multiple risk factors for candidiasis, including recent abdominal surgery, anastomotic leak, or necrotizing pancreatitis, who are doing poorly despite treatment for bacterial infection [95].

Guidelines recommend echinocandins as first-line treatment in for invasive candidiasis [94, 95]. However, their role has been debated. Antifungal resistance is a growing concern in *Candida* spp [96]. . Moreover, echinocandin exposure has been reported to be suboptimal in critically ill patients. Dose adjustments supported by therapeutic drug monitoring (TDM) (if available) are suggested in treating patients with intra-abdominal candidiasis [97]. The PK and antifungal activity of the three echinocandins: anidulafungin, micafungin, and caspofungin were assessed in ascites fluid and plasma of critically ill adults treated for suspected or proved invasive candidiasis [98], showing that standard daily doses of anidulafungin, micafungin, and caspofungin may result in ascites fluid concentrations that inhibit proliferation of *C. albicans* and *C. glabrata*, but are not fungicidal.

Azole antifungal agents are not recommended as empiric therapy because activity against non-*albicans Candida* spp. is not universal. Isavuconazole penetration into ascites fluid is variable [99]. Overall success of treatment with isavuconazole in cases of intra-abdominal candidiasis depends on the interplay of the susceptibility of the isolate, the immune status of the host, and possibly other factors yet to be elucidated. Whereas azoles are not considered first-choice therapy due to the possibility of resistance and numerous drug-drug interactions (especially with fluconazole), an alternative may be lipid formulations of amphotericin B [100]. The IDSA guidelines recommend the use of a standard-dose echinocandin as initial therapy, and a lipid formulation of amphotericin B (3–5 mg/kg daily) for patients with suspected azole- and echinocandin-resistant *Candida* infections [95]. The ESICM/ESCMID guidelines make a “strong” recommendation for the use of echinocandins and a “moderate” strength recommendation for lipid-formulation amphotericin B (L-amB) [94]. Recently, a single 5 mg/kg/kg administration of L-amB whilst waiting for the result of 1,3-beta-D-glucan testing was reported to be safe and cost-effective in a single-center experience [101].

#### Clinical conditions

### Statement 17

**Sepsis and septic shock are time-dependent emergencies and resuscitation should start immediately (High-quality evidence, Strong recommendation).**

### Statement 18

**Fluid administration should be individualized for every patient, based on the evaluation of a need for fluid and on any premorbid conditions (Low-quality evidence, strong recommendation).**

The WISS study [1] showed that mortality was increased significantly by sepsis and that mortality was increased further in patients who developed severe sepsis or septic shock. Sepsis-related mortality was as follows: no sepsis, 1.2%; sepsis, 4.4%; severe sepsis, 27.8%; septic shock, 67.8%. Since that time, clinical severity grading of patients with sepsis has been re-defined by SEPSIS-3 [102, 103], which eliminated “severe sepsis” as a redundant category and re-defined “sepsis” as infection associated with organ dysfunction. Under SEPSIS-3, sepsis is defined as life-threatening organ dysfunction due to a dysregulated host response, stressing the potential lethality of this condition and the need for urgent recognition and intervention. Organ dysfunction is defined as an increase in the Sequential Organ Failure Assessment (SOFA) score of 2 points or more [104], whereas septic shock is characterised by a vasopressor requirement to maintain mean arterial pressure (mAP) *≥* 65 mm Hg and a serum lactate concentration < 2 mmol/L (> 18 mg/dL) after restoration of euvolemia. Human immune responses to infection can vary among individuals. Some patients can “overreact” by generating a massive “cytokine storm,” whereas other patients may be less reactive, or can restore homeostasis promptly, appearing to have a lesser response to a similar infection. Prompt identification of patients for resuscitation and surgical intervention substitutes for deranged or failing host responses, particularly of the peritoneal cavity, and may improve patient outcomes via prompt restoration of homeostasis [105, 106] and maintenance of peritoneal toilet.

Prompt intravenous fluid administration is mandatory in patients with sepsis and septic shock.

Two randomized single-center trials [107, 108] compared balanced crystalloid solution to isotonic saline in critically ill adults, finding that mortality, new-onset kidney replacement therapy, and persistent kidney dysfunction were each lower with balanced crystalloids. Although albumin is theoretically more efficient than crystalloid in sustaining oncotic pressure, it is more expensive and there is no clear benefit to its use [106].

### Statement 19

**Vasopressor agents should be administered to restore organ perfusion as soon as possible if blood pressure is not restored after initial fluid resuscitation (Low-quality evidence, Strong recommendation).**

If fluid resuscitation fails or is protracted, vasopressor agents should be administered to restore organ perfusion, maintaining but usually not exceeding the aforementioned target for mAP. The initial vasopressor of choice is norepinephrine, which has a beta-agonist effect at low doses but is increasingly an alpha-agonist at escalating doses [109, 110]. Although most patients in shock show improvement in hemodynamics after starting norepinephrine, a proportion of patients remains with a poor clinical response to catecholamines, e.g., requiring large doses (> 0.5 mcg/Kg/min of norepinephrine) to achieve mAP of 65 mm Hg, if the threshold is reached at all [111]. In refractory cases, addition of a second vasopressor may be advantageous rather than increasing further the norepinephrine dose. Low-dose arginine vasopressin (0.03–0.06 IU/min by continuous infusion) has a “catecholamine-sparing” effect, and also reduces mortality [112].

### Statement 20

**Fluid overload should be avoided in adults with sepsis or septic shock of abdominal origin (Very low-quality evidence, Strong recommendation).**

To avoid an adverse outcome, fluid overload should be avoided in adults with sepsis or septic shock of abdominal origin for several reasons. Especially for patients requiring urgent surgical intervention, overly aggressive fluid resuscitation may increase intra-abdominal pressure and heighten the inflammatory response, which is associated with a higher risk of complications [113]. The systemic inflammatory response, increased vascular permeability, and aggressive fluid resuscitation predispose to intralumenal and extravascular fluid sequestration, ascites formation, and bowel wall edema. These changes, if the abdominal wall has been closed, may result in intra-abdominal hypertension (IAH) and abdominal compartment syndrome (compromised organ function, primarily respiratory and renal).

### Statement 21

**Specific adjunctive therapy can be considered in selected patients, evaluating the potential benefit and possible harm. (Low quality evidence, Weak recommendation)**

In sepsis, mortality is higher when both pro- and anti-inflammatory cytokine concentrations are elevated [114, 115]. The rationale of using extracorporeal blood purification in patients with septic shock is twofold: Clearance of bacterial lipopolysaccharides or other mediators, and modulation of the immune response. Blood purification for sepsis has consisted of various techniques: High-volume hemofiltration, high-adsorption hemofiltration, high cut-off membrane hemofiltration, plasma exchange, and hybrid systems [116]. A systematic review and meta-analysis of RCTs between various blood purification techniques and all-cause mortality in human beings with sepsis [117] included 10 single- and 6 multicenter studies; techniques used included hemoperfusion (10 studies), hemofiltration (4 studies), and plasma exchange (2 studies). Overall, blood purification decreased mortality, but results were driven mainly by hemoperfusion in trials conducted in Japan.

Polymyxin B hemoperfusion (PMB-HP) is controversial [118, 119]. A French multi-center RCT (ABDOMIX) enrolled 243 patients with septic shock within 12 h after emergency surgery for perforated viscus [120], but no benefit was demonstrated. The EUPHRATES RCT of PMB-HP in patients with septic shock and confirmed endotoxemia also failed to show improved survival [121]. However, some patients with septic shock had extremely high burdens of endotoxin activity (EA ≥ 0.9). In a post-hoc analysis of the EUPHRATES trial evaluating 194 patients with EA between 0.60 and 0.89 who completed two treatments (active or sham), at 28 days, 23/88 patients (26.1%) in the active group died *versus* 39/106 (36.8%) in the sham group. Twenty-eight-day survival of the active group was longer than survival of the sham group (HR 0.56, 95% CI 0.33–0.95, *p* = 0.03] [122]. The in-progress TIGRIS RCT of endotoxic shock (EA 0.60–0.89) (Clinical Trials.gov identifier: NCT03901807) may be more informative.

Intravenous immunoglobulin (IVIg), another mechanism for neutralization of endotoxin and other bacterial toxins [123] is controversial for surgical sepsis. In a meta-analysis of the clinical effectiveness of IVIg [123] (18 RCTs), IVIg reduced sepsis mortality, but low study quality, heterogeneous Ig preparations and dosing regimens, and different comparators (placebo *versus* albumin) confounded interpretation. The available evidence is insufficient to support the widespread use of IVIg as therapy for sepsis.

### Statement 22

**For adults with septic shock, early and properly administered empiric antibiotic therapy has a significant impact on outcome (Moderate-quality evidence, Strong recommendation).**

Early, appropriate empiric antibiotic therapy for septic shock can have a substantial beneficial impact on outcome, independent of the anatomic origin of infection [124]. Given the high risk of death from septic shock and the strong association of delayed antibiotic therapy with mortality, prompt antibiotic administration is crucial. However, for sepsis without shock, the association between time to antibiotic administration and mortality is less pronounced [125, 126]. Therefore, absent shock and if source control is adequate, it may be possible to defer antibiotic therapy until culture and susceptibility testing can inform targeted therapy [126].

### Statement 23

**In adults with sepsis or septic shock, appropriate dosing and administration of antibiotics should include a loading dose and extended or continuous infusion for beta-lactam agents. (Low-quality evidence, Strong recommendation)**

Critically ill patients with sepsis or septic shock may exhibit altered antibiotic PK. Therefore, higher-than-standard loading doses of beta-lactam agents should be considered regardless of renal function [127]. Once the regimen is established, it is important to reassess daily, because fluctuating fluid balance and organ function may affect antibiotic PK. In critically ill patients, plasma creatinine is an unreliable marker of renal function. For beta-lactams, appropriate dosing supports time-dependent bactericidal activity. Beta-lactam antibiotic bactericidal activity is optimal when antibiotic concentrations are maintained (just) above the MIC of the pathogen for at least 70% of the dosing interval (fT *≥* 70%) [129]. For beta-lactam agents, prolonged or continuous infusions optimize fT > MIC [128–131].

Tissue distribution is another important aspect to consider in prescribing antibiotics because high concentrations at the infection site may prevent resistance development. Tissue distribution is higher for lipophilic than hydrophilic agents, but disease-related factors (e.g., binding to plasma proteins, serum albumin concentration) may influence tissue distribution. In a 2020 prospective observational study of critically ill patients with cIAI who required surgery and received empiric beta-lactam antibiotic therapy [132] high doses of beta-lactams ensured 100% fT > 4×MIC for 78% of patients within the first 24 h. TDM of beta-lactams, as a dose optimization and individualization tool, has been recommended, but despite potential benefit, not all centers perform TDM for beta-lactams [133].

### Statement 24

**After rapid patient stabilization, adults with sepsis and septic shock should undergo a source control procedure within 6 h**. **Time from admission to initiation of surgery for source control is a crucial determinant of survival in from sepsis and septic shock (Low-quality evidence, Strong recommendation).**

Comprehensive knowledge of disease and sepsis physiopathology, the range of surgical and nonsurgical options, and how to balance benefit and risk are all necessary to devise a treatment plan and achieve source control as soon as possible following resuscitation. Limited data suggest that source control should be obtained within six hours for optimal results. Bloos et al. [134] demonstrated that the median time from onset of severe sepsis or septic shock to source control for 1,011 patients from 44 German ICUs was 2 h for survivors and 5.7 h for non-survivors. Time to source control > 6 h was independently associated with increased mortality (as were age and disease severity). A 2017 trial [135] demonstrated that achievement of surgical source control was significantly related to 28-day mortality, with 1% increased mortality per hour of delay to source control. A 2014 prospective observational study of 154 patients with GI perforation [136] demonstrated that each hour of delay correlated with decreased survival, with the target time for a favourable outcome within 6 h from admission. A 2022 post-hoc analysis of a multi-center observational study (Abdominal Sepsis Study, AbSeS) of 2,621 adult ICU patients with cIAIs (306 ICUs, 42 countries) [137] included 1,077 cases of microbiology-confirmed secondary peritonitis. Mortality was 29.7%. A stepwise increase in mortality was observed with increasing SOFA scores. The highest odds of death were associated with septic shock, late-onset hospital-acquired peritonitis, and failed source control. Compared with “emergency” source control intervention (within < 2 h of diagnosis), “urgent” source control was the only modifiable covariate associated with lower odds of mortality.

Arguably the strongest available evidence for achieving source control within 6 h comes from a planned post-hoc analysis of the MEDUSA trial [138], including forty German hospitals, 4,792 patients who received antimicrobial therapy, and 1,595 patients who underwent surgical source control. 28-day mortality increased by 0.42% for each hour of delay, and was significant in patients with and without shock. Delays > 6 h significantly increased mortality. Each hour of delay in antimicrobial therapy also increased the risk of progression from sepsis to shock. However, time to surgical source control was not associated with decreased odds of successful source control or increased odds of death when adjusted for confounders. Only among septic shock patients was delay of source control significantly related to mortality.

### Statement 25

**For adults with abdominal sepsis or septic shock, on-demand re-laparotomy (as opposed to scheduled reoperation) should be the first-choice approach to re-operation (Moderate quality evidence, Strong recommendation).**

### Statement 26

**Damage control surgery may be an option in selected physiologically deranged patients with sepsis of abdominal origin (Very low-quality evidence, Strong recommendation).**

### Statement 27

**Based on the complexity of IAI (variable clinical presentations depending on the original site of infection, the causative organism, the pattern of acute organ dysfunction, and the underlying health status of the patient), an individualized, carefully designed approach to source control is the best pathway to follow (Very low-quality evidence, Strong recommendation).**

After successful implementation in trauma, damage control laparotomy (DCL) has been used increasingly to manage non-traumatic emergencies, including critically ill patients with cIAIs [139]. DCL for cIAI, sepsis, and critical illness, meaning abbreviated laparotomy with subsequent reoperation for delayed definitive repair after physiologic resuscitation is completed [140], can be lifesaving. DCS is linked closely to the “open abdomen” approach, which provides manual peritoneal toilet while intraperitoneal host defenses recover from the infectious insult. The open abdomen, plicated with an easily removed/replaced temporary abdominal closure device, facilitates early identification and drainage of any residual infection and removal of peritoneal fluid, reducing the risk of abdominal compartment syndrome. Definitive intervention and anastomosis are deferred until the patient is resuscitated, vital signs and hemodynamics are normal, and peritoneal contamination is controlled. However, open-abdomen techniques may require multiple returns to the operating room, or bedside laparotomy in the ICU. The potential downside of repetitive DCL is substantive potential complications, including “entero-atmospheric” fistula, loss of abdominal wall domain, and large abdominal wall hernias, encouraging early (within 7 days) abdominal wall reconstruction to prevent those complications. The COOL study may clarify the role of open abdomen management in the setting of sepsis of abdominal origin [141].

#### Host immune status

### Statement 28

**Immunocompromised patients with IAIs are at increased risk of morbidity and mortality and may fail standard NOM when otherwise indicated. (Low-quality evidence, Strong recommendation)**

Host immune status is important to consider, but difficult to quantify. Immunocompromised patients represent heterogeneous states that include congenital conditions (T- or B-cell defects or macrophage dysfunction), and can afflict newborns, children, and adults. Acquired conditions include human immunodeficiency virus/acquired immunodeficiency syndrome, patients with malignant liquid or solid tumors undergoing chemotherapy [142], solid-organ transplant (SOT) recipients, or patients with inflammatory or rheumatologic disease treated with immunomodulators [143]. Notably, immune checkpoint inhibitor therapy has been associated rarely with the development of acute appendicitis [144].

Diagnosis and treatment in immunocompromised patients can be challenging. Immunocompromised patients with IAIs are at increased risk of morbidity and mortality [145–147], and may fail standard NOM when otherwise indicated. As such, most patients require surgical intervention. Recent multi-society source control guidelines [9] proposed a categorization of patients into 3 classes according to current condition, comorbidities, and ongoing therapies (e.g., anticoagulants or steroids), together with their immunologic state (Table 1).

**Table 1:** Proposed Patient Classification for Comorbidities and Immunocompromise [9]

Patient stratification	
CLASS A	Healthy patients with none or well-controlled comorbidities, and no immunocompromise, where the infection is the principal problem.
CLASS B	Patients with major comorbidities or moderate immunocompromise, but stable clinically, in whom the infection, without control, can worsen the prognosis rapidly.
CLASS C	Patients with major comorbidities in advanced stages or severe immunocompromise, in which the infection worsens an already severe clinical condition.

SOT is an established option for patients with end-stage organ dysfunction. Patient survival has increased in recent years due to improved surgical techniques, perioperative management, immunosuppressive agents and tactics, and anti-infective prophylaxis to prevent opportunistic infection. Although the incidence of post-SOT cytomegalovirus infection has decreased, infections linked to MDR gram-negative bacteria are increasing. Considering that SOT recipients are exposed frequently to antibiotics in the healthcare setting [148], the vulnerability of SOT recipients to MDR infections is a crucial determinant of decisions to start antimicrobial therapy.

## Conclusions

cIAI, a condition that is sometimes difficult to manage, requires more than antimicrobial therapy to guide the treatment of the patient, even in the era of AMR. Therapy should be individualized if possible. The anatomic extent of infection, the presumed pathogens involved and risk factors for AMR, the origin of the infection, the patient’s clinical condition, the host’s immune status, and available treatment options should be always assessed to optimize the management of patients with cIAIs.

## Data Availability

No datasets were generated or analysed during the current study.

## Abbreviations

AAC: Acute Acalculous Cholecystitis
AMR: Antimicrobial Resistance
APACHE: Acute Physiology and Chronic Health Evaluation
CDC: Centers for Disease Control and Prevention
CI: Confidence Interval
cIAIs: Complicated Intra-abdominal Infections
CRE: Carbapenem-Resistant Enterobacterales
CT: Computed Tomography
DAP: Device-Associated Peritonitis
DCL: Damage Control Laparotomy
ECDC: European Centre for Disease Prevention and Control
ESBLs: Extended-Spectrum Beta-Lactamases
ESCMID: European Society of Clinical Microbiology and Infectious Diseases
ESICM: European Society of Intensive Care Medicine
GI: Gastrointestinal
GRADE: Grading of Recommendations Assessment, Development, and Evaluation
HR: Hazard Ratio
IAH: Intra-Abdominal Hypertension
IAIs: Intra-Abdominal Infections
ICU: Intensive Care Unit
IDSA: Infectious Disease Society of America
IVIg: Intravenous Immunoglobulin
KPC: K. pneumoniae Carbapenemase
mAP: Mean Arterial Pressure
MBLs: Metallo-Beta-Lactamases
MDR: Multidrug-Resistant
MIC: Minimal Inhibitory Concentration
NOM: Non-Operative Management
OR: Odds Ratio
PCT: Procalcitonin
PDR: Pan Drug-Resistant
PID: Pelvic Inflammatory Disease
PK: Pharmacokinetics
PMB-HP: Polymyxin B Hemoperfusion
PP: Post-operative Peritonitis
RCT: Randomized Controlled Trial
SOFA: Sequential Organ Failure Assessment
SOT: Solid-Organ Transplant
TDM: Therapeutic Drug Monitoring
TOC: Test-Of-Cure
uIAIs: Uncomplicated Intra-Abdominal Infections
US: Ultrasonography
XDR: Extensively Drug-Resistant
